# Sulforaphane Protects Against Ethanol-Induced Apoptosis in Human Neural Crest Cells Through Diminishing Ethanol-Induced Hypermethylation at the Promoters of the Genes Encoding the Inhibitor of Apoptosis Proteins

**DOI:** 10.3389/fcell.2021.622152

**Published:** 2021-02-09

**Authors:** Yihong Li, Huadong Fan, Fuqiang Yuan, Lanhai Lu, Jie Liu, Wenke Feng, Huang-Ge Zhang, Shao-Yu Chen

**Affiliations:** ^1^Department of Pharmacology and Toxicology, University of Louisville Health Science Center, Louisville, KY, United States; ^2^University of Louisville Alcohol Research Center, Louisville, KY, United States; ^3^Department of Medicine, University of Louisville, Louisville, KY, United States; ^4^Department of Microbiology and Immunology, James Graham Brown Cancer Center, University of Louisville, Louisville, KY, United States; ^5^Robley Rex Veterans Affairs Medical Center, Louisville, KY, United States

**Keywords:** sulforaphane, ethanol, apoptosis, DNA methylation, IAP proteins, neural crest cells

## Abstract

The neural crest cell (NCC) is a multipotent progenitor cell population that is sensitive to ethanol and is implicated in the Fetal Alcohol Spectrum Disorders (FASD). Studies have shown that sulforaphane (SFN) can prevent ethanol-induced apoptosis in NCCs. This study aims to investigate whether ethanol exposure can induce apoptosis in human NCCs (hNCCs) through epigenetically suppressing the expression of anti-apoptotic genes and whether SFN can restore the expression of anti-apoptotic genes and prevent apoptosis in ethanol-exposed hNCCs. We found that ethanol exposure resulted in a significant increase in the expression of DNMT3a and the activity of DNMTs. SFN treatment diminished the ethanol-induced upregulation of DNMT3a and dramatically reduced the activity of DNMTs in ethanol-exposed hNCCs. We also found that ethanol exposure induced hypermethylation at the promoter regions of two inhibitor of apoptosis proteins (IAP), NAIP and XIAP, in hNCCs, which were prevented by co-treatment with SFN. SFN treatment also significantly diminished ethanol-induced downregulation of NAIP and XIAP in hNCCs. The knockdown of DNMT3a significantly enhanced the effects of SFN on preventing the ethanol-induced repression of NAIP and XIAP and apoptosis in hNCCs. These results demonstrate that SFN can prevent ethanol-induced apoptosis in hNCCs by preventing ethanol-induced hypermethylation at the promoter regions of the genes encoding the IAP proteins and diminishing ethanol-induced repression of NAIP and XIAP through modulating DNMT3a expression and DNMT activity.

## Introduction

Prenatal alcohol exposure can cause a spectrum of physical abnormalities and mental dysfunctions in children, which are defined as Fetal Alcohol Spectrum Disorders (FASD) (Koren et al., 2003; Sokol et al., 2003). Previous studies by Kotch and Sulik, as well as Dunty et al., have revealed that ethanol exposure during the early stages of development resulted in excessive cell death in neural crest cells (NCCs), which contributes heavily to ethanol-induced malformations (Kotch and Sulik, 1992; Dunty et al., 2001). Using a chick-specific antibody to NCCs, Cartwright and Smith confirmed that the prenatal ethanol exposure resulted in the loss of cranial NCCs in chicken embryos (Cartwright and Smith, 1995). Studies from our laboratory have also shown that ethanol exposure significantly increased apoptosis in NCCs *in vitro* (Chen et al., 2015; Li et al., 2019b).

Multiple signaling pathways have been shown to be involved in the ethanol-induced apoptosis in NCCs, including the Bcl2 family (Chen et al., 2015; Yuan et al., 2018), Seven *in absentia* homolog1 (Siah1) (Sun et al., 2014; Yuan et al., 2017), p53 and MAPK signaling (Yuan et al., 2017, 2020) and Nrf2 signaling (Chen et al., 2013). However, the potential role of the inhibitor of apoptosis proteins (IAP) in ethanol-induced apoptosis in NCCs remains to be defined. IAP family is composed of eight members presenting one to three BIR domains from the baculovirus (Birnbaum et al., 1994). They are frequently overexpressed in cancer cells that are resistant to apoptosis (Silke and Meier, 2013). DNA hypomethylation at the promoters of IAP genes and the overexpression of the IAPs have been found in different cancers, including ovarian, hepatocellular carcinoma (HCC), and glioma (Hervouet et al., 2013). Studies have also shown that the loss of methylation induced by DNA methyltransferase (DNMT) inhibitor at the promoters of the IAP genes is associated with apoptosis resistance (Hervouet et al., 2013).

It is well-known that DNA hypermethylation in the promoter regions of genes is associated with gene repression (Jones and Baylin, 2002) and plays an important role in the repression of many genes involved in various cellular functions, including DNA repair, cell adhesion, and apoptosis (Teodoridis et al., 2004; Gopisetty et al., 2006). DNA methylation has been shown to be associated with the repression of the apoptotic genes in cancer cells (Murphy et al., 2008; Malekzadeh et al., 2009; Hervouet et al., 2013). Hypermethylation at the *Casp 8* promoter was correlated with a low Casp 8 expression in cancer cells and contributed to apoptosis resistance in different cancer cell lines, such as pediatric cancer (Harada et al., 2002), lung carcinomas (Shivapurkar et al., 2002), and breast cancer (Wu et al., 2010). Studies have shown that ethanol exposure can alter DNA methylation (Haycock and Ramsay, 2009; Ouko et al., 2009; Zhou et al., 2011) and histone modification (Zhong et al., 2010; Bekdash et al., 2013; Subbanna et al., 2013, 2014). An earlier study by Garro has also shown that fetal DNA was hypermethylated after ethanol exposure during embryonic development (Garro et al., 1991).

DNA methylation occurs on the cytosine residue of CpG dinucleotides through the transfer of 5-methylcytosine from the methyl donor S-adenosylmethionine (SAM) to the CpG. The reaction is catalyzed by a family of enzymes called DNA methyltransferases (DNMTs), including DNMT1, DNMT3a, and DNMT3b (Singal and Ginder, 1999). DNMT1 is responsible for the maintenance of established patterns of DNA methylation, while DNMT3a and DNMT3b mediate the establishment of new or *de novo* DNA methylation patterns (Chen et al., 2003; Jones and Liang, 2009b). Alteration of DNA methylation by DNMTs may trigger hypermethylation or hypomethylation of gene promoters and consequently result in activating or inhibiting gene expression (Hervouet et al., 2010, 2013).

Sulforaphane (SFN) is a vegetable-derived isothiocyanate that is abundant in cruciferous vegetables such as broccoli. Our previous studies have shown that SFN prevented ethanol-induced apoptosis through upregulating the antioxidant gene Nrf2 in NCCs (Chen et al., 2013). In addition to acting as an Nrf2 inducer, SFN has been found to regulate gene expression by inhibiting the activity of DNMTs (Meeran et al., 2012). It has also been reported that SFN can inhibit LPS-induced DNMT3a gene expression and confer resistance to LPS-induced apoptosis in porcine monocyte-derived dendritic cells (Qu et al., 2015).

In the present study, we determined whether SFN can prevent ethanol-induced apoptosis in hNCCs through epigenetic modulation of anti-apoptotic genes. We found that ethanol exposure resulted in a significant increase in the expression of DNMT3a and the activity of DNMTs. SFN treatment diminished the ethanol-induced upregulation of DNMT3a and dramatically reduced the activity of DNMTs in ethanol-exposed hNCCs. Ethanol exposure also induced hypermethylation at the promoter regions of two IAP proteins, NAIP and XIAP, in hNCCs, which were prevented by co-treatment with SFN. SFN treatment also significantly diminished ethanol-induced downregulation of NAIP and XIAP in hNCCs. The knockdown of DNMT3a significantly enhanced the effects of SFN on preventing the ethanol-induced repression of NAIP and XIAP and apoptosis in hNCCs. These results demonstrate that SFN can prevent ethanol-induced apoptosis in hNCCs by diminishing the ethanol-induced hypermethylation at the promoter regions of the genes encoding the IAP proteins.

## Materials and Methods

### Human Neural Crest Cell Differentiation, Culture and Treatment

hNCCs were differentiated from human embryonic stem cell (hESC) line H9 (WA09)), which was purchased from WiCell^®^ (Madison, WI, United States). hESCs were maintained in mTeSR^TM^1 (StemCell Technologies, Inc., Vancouver, Canada) on hESCs-qualified Matrigel^TM^ (BD Biosciences, San Jose, CA) coated plates, following the WiCell’s protocols. The differentiation of hNCCs from hESCs was performed as previously reported (Menendez et al., 2013; Avery and Dalton, 2016) with modification. Briefly, the hESCs^®^ were first adapted to Accutase solution (Stem Cell Technologies, Vancouver, BC, Canada) and dissociated into single cells and cultured in the hESCs maintenance medium. When hESCs reached 75–85% confluence, the hESCs maintenance medium was removed, and the hESCs were detached by adding the Accutase^®^ solution. The collected hESCs were resuspended and cultured in the hNCCs differentiation medium (DMEM/F-12 Medium, 14.3 M L-Glutamine + β-mercaptoethanol, MEM Non-Essential Amino Acid, 10 μg/mL Fgf2, 10 μg/mL Heregulin β-1, 200 μg/mL Long R3-IGF1, 10 mM CHIR 99021, 10 mM SB421542 and Penicillin and streptomycin). When cells reached 75–85% confluence, the differentiating cells were passed using Accutase^®^ and then maintained in hNCCs differentiation medium. After culture in hNCCs-differentiation medium for around 10 days, hNCCs were collected, and NCC identity was analyzed by examining the NCC markers p75, HNK1, and AP2 using immunocytochemistry, flow cytometry and/or RT-PCR. For treatments, hNCCs were first pretreated with 1 μM SFN (LST Laboratories, St. Paul, MN) for 24 h, and then the cells were concurrently exposed to 1 μM SFN and 50 mM ethanol for an additional 24 h. To maintain the stable ethanol levels, the cell culture dishes were placed in a plastic desiccator containing ethanol in distilled water, as described previously (Yan et al., 2010).

### Quantitative Real-Time PCR

Quantitative real-time PCR was performed as previously described (Fan et al., 2019). Briefly, total RNA was isolated from control and treated hNCCs using a QIAGEN RNeasy mini kit (QIAGEN, Valencia, CA) according to the manufacturer’s instruction. The cDNAs were synthesized using QuantiTect Reverse Transcription Kit (QIAGEN, Valencia, CA) following the manufacturer’s protocol. Quantitative RT-PCR was performed using FastStart SYBR Green Master (Roche Applied, Indianapolis, IN, United States) on a Rotor-Gene 600 Real-time PCR system (Corbett LifeScience, Mannheim, Germany). The following primer pairs were used for this analysis: NAIP (Homo sapiens) forward: 5′-GCCTAGATGCAGTTCAGTTGG-3′, reverse: 3′-GGCACCAAAGAGGATTAGGCT-3′; XIAP (Homo sapiens) forward: 5′-GGTGAAGGAGATACCGTGCG-3′, reverse: 3′-GCATGTGTCTCAGATGGCCT-5′; β-Actin (Homo sapiens) forward: 5′-AGAAGGATTCCTATGTGGGCG-3′, reverse: 3′-GGATAGCACAGCCTGGATAGCA-5′. The primers were synthesized by Integrated DNA Technologies, Inc. (IDT, Coralville, IA, United States). All assays were carried out in triplicate. Relative quantitative analysis was performed by comparing the threshold cycle number for target genes and a reference β-Actin mRNA.

### Western Blotting

Western blotting was performed by the standard protocol as previously described (Yuan et al., 2017). Briefly, hNCCs from control and experimental groups were washed with PBS and then lysed in cold RIPA lysis buffer with 1 mM PMSF and protease cocktail inhibitors. Whole-cell lysis was centrifuged at 12,000 × *g* for 10 min at 4°C, and the supernatants were used for Western blot. Protein concentrations were measured by using BSA Protein Assay Reagent Kit (Pierce, Thermo Scientific, Waltham, MA). Proteins were probed with the following antibodies: anti-cleaved caspase-3 rabbit mAb (Cell Signaling, Beverly, MA, United States), anti-DNMT3a rabbit mAb (Cell Signaling, Beverly, MA, United States), anti-NAIP sheep pAb (R&D system, Minneapolis, MN, United States), anti-XIAP rabbit mAb (Cell Signaling, Beverly, MA, United States), and anti-β–Actin mouse mAb (Santa Cruz, Santa Cruz, CA). The densitometry of the blots was analyzed using ImageJ software (National Institute of Health, United States). All Western blot analyses were performed in triplicate.

### Analysis of DNMT Activity

hNCCs from the control and treated groups were harvested, and nuclear extracts were isolated by using the EpiQuik Nuclear Extraction Kit (Epigentek, Farmingdale, NY) according to the manufacturer’s protocol. The protein concentrations of nuclear extraction were measured using the BCA protein assay kit (Thermo Scientific, Irvine, CA). DNMT activity was determined with an EpiQuik DNA Methyltransferase Activity Assay kit (Epigentek, Farmingdale, NY), following the manufacturer’s instruction.

### Methylated Specific PCR (MSP)

The methylation status of CpG island on the promoters of NAIP and XAIP was determined by MSP. Genomic DNA was extracted from control and treated hNCCs using DNeasy Blood & Tissue Kit (QIAGEN, Valencia, CA) according to the manufacturer’s instruction. Bisulfite conversion of the genomic DNA was performed using the EpiTect Bisulfite Kit (QIAGEN, Valencia, CA). Bisulfite-modified DNA was amplified using two primer sets specific for the detection of the methylated (M) and unmethylated (U) sequences. The following primer pairs were used for MSP analysis. NAIP (M) forward: 5′-AGGTTGGAGTATCGTGGC-3′, reverse: 3′-GAACGTAATAACGAACGCCT-5′, NAIP (U) forward: 5′-TTTAGGTTGGAGTATTGTGGT-3′, reverse: 3′-ACCAAACATAATAACAAACACCT-5′; XIAP (M) forward: 5′-GGCGGAGGTTGTAGTGAGTC-3′, reverse: 3′-ATAAACATAAACCACCGCGC-5′, XIAP (U) forward: 5′-GGAGGTGGAGGTTGTAGTGAGTT-3′, reverse: 3′-TATAAACATAAACCACCACACCC-5′. Real-time qMSP assays were performed on a Rotor-Gene 600 Real-time PCR system (Corbett LifeScience, Mannheim, Germany), and the PCR products were separated on 2% agarose gel. The intensity of DNA bands was measured by using NIH ImageJ software (National Institute of Health, United States). Methylation levels were calculated as the intensity of the methylated DNA band relative to the combined intensity of the methylated and unmethylated DNA bands.

### siRNA Transfection

hNCCs were transfected with DNMT3a siRNA (SMART pool: On-TARGET plus human DNMT3a) (GE Healthcare Dharmacon, Lafayette, CO) or scramble control siRNA (IDT, Coralville, IA) in a final concentration of 25 nM by using Lipofectamine^TM^2000 (Thermo Fisher, Waltham, MA), according to the manufacturer’s instruction. The cells were harvested 24 h after transfection for experiments.

### Analysis of Cell Viability and Apoptosis

Cell viability was measured using an MTS (3-(4,5-dimethylthiazol-2-yl)-5-(3- arboxymethoxyphenyl)-2-(4- sulfophenyl)-2*H*-tetrazolium salt) assay kit (Promega, Madison, WI), as described previously (Chen et al., 2013). Apoptosis was determined by the analysis of caspase-3 cleavage by Western blot and by flow cytometry using a FITC Annexin V apoptosis detection kit (BD Bioscience, Franklin Lakes, NJ, United States) as described previously (Li et al., 2019b).

### Statistical Analysis

Statistical analyses were performed using GraphPad Prism software (GraphPad Software, San Diego, CA, United States). All data were expressed as mean ± SEM of at least three independent experiments. Comparisons between groups were analyzed by one-way ANOVA. Multiple comparison post-tests were conducted using Bonferroni’s test. Differences between groups were considered significant at *p* < 0.05.

## Results

### SFN Treatment Significantly Diminished Ethanol-Induced Apoptosis in hNCCs

**FIGURE 1:**
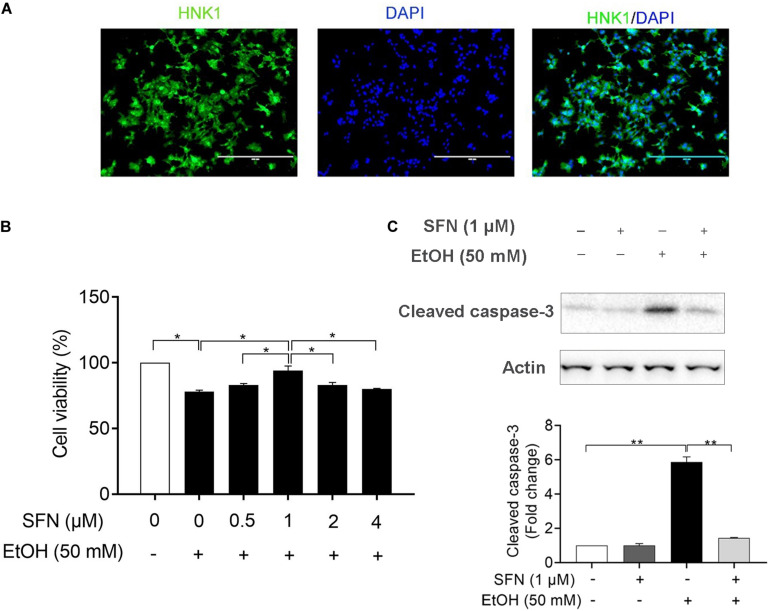
SFN significantly diminished the ethanol-induced apoptosis in hNCCs. **(A)** Immunocytochemistry of NCC marker, HNK1, in hNCCs differentiated from hESCs. **(B)** hNCCs were treated with 50 mM ethanol alone or pretreated with SFN at different concentrations for 24 h, followed by concurrent exposure to SFN and ethanol for an additional 24 h. Cell viability was determined by MTS assay. **(C)** hNCCs were treated with 1 μM SFN for 24 h, followed by concurrent exposure to 1 μM SFN and 50 mM ethanol for an additional 24 h. Apoptosis was determined by the analysis of caspase-3 cleavage using Western blotting. Data are expressed as a percentage of control **(B)** or fold change over control **(C)** and represent the mean ± SEM of three separated experiments. *p < 0.05, **p < 0.01.

For this study, hNCCs were differentiated from human embryonic stem cells and validated by examining the expression of NCC marker HNK1 using immunocytochemistry (Figure 1A). To determine whether SFN treatment can diminish the ethanol-induced reduction in cell viability in hNCCs, the cells were treated with ethanol alone or pretreated with SFN at different concentrations for 24 h, followed by concurrent exposure to SFN and ethanol for an additional 24 h. As shown in Figure 1B, SFN at 1 μM significantly diminished the reduction of cell viability in hNCCs exposed to 50 mM ethanol. Treatment with SFN also significantly reduced caspase-3 activation in hNCCs exposed to 50 mM ethanol for 24 h (Figure 1C).

### SFN Diminished Ethanol-Induced Increase in DNMT Activity and Up-Regulation of DNMT3a in hNCCs

**FIGURE 2:**
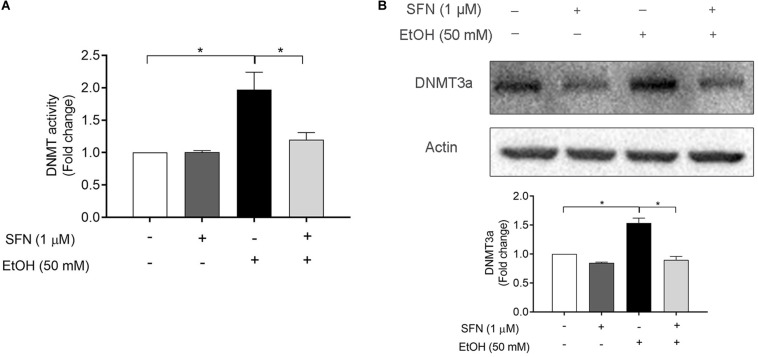
SFN diminished ethanol-induced increase in DNMT activity and the up-regulation of DNMT3a in hNCCs. hNCCs were treated with 1 μM SFN for 24 h, followed by concurrent exposure to 1 μM SFN and 50 mM ethanol for an additional 24 h. **(A)** The DNMT activity was determined by using an EpiQuik DNA Methyltransferase Activity Assay kit. **(B)** The protein expression of DNMT3a was determined by Western blotting. Data are expressed as fold change over control and represent the mean ± SEM of three separated experiments. *p < 0.05.

To determine the effects of ethanol and SFN on the activity and expression of DNMT, hNCCs were pretreated with 1 μM SFN for 24 h, followed by 24 h of concurrent exposure to 1 μM SFN and 50 mM ethanol. As shown in Figure 2A, ethanol exposure significantly increased the DNMT activity in hNCCs. Treatment with SFN dramatically reduced the ethanol-induced increase in the activity of DNMT in hNCCs. To determine whether ethanol and SFN also affect the expression of DNMT, the protein expression of DNMT3a, one of the three DNMTs, which is involved in DNA methylation in eukaryotic cells (Leonhardt and Bestor, 1993; Jones and Liang, 2009a), was analyzed by Western blotting. We found that exposure to 50 mM ethanol resulted in a significant increase in the protein expression of DNMT3a in hNCCs. Co-treatment with SFN and ethanol significantly reduced ethanol-induced increase in DNMT3a protein expression (Figure 2B).

### SFN Diminished the Ethanol-Induced Hypermethylation at the Promoters of NAIP and XIAP in hNCCs

**FIGURE 3:**
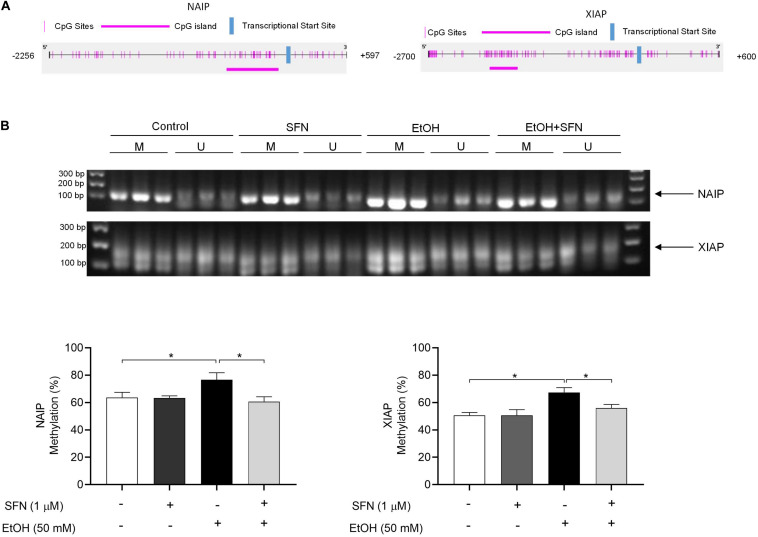
SFN significantly diminished the ethanol-induced hypermethylation at the promoters of XIAP and NAIP in hNCCs. hNCCs were treated with 1 μM SFN for 24 h, followed by concurrent exposure to 1 μM SFN and 50 mM ethanol for an additional 24 h. **(A)** Schematic illustration of the CpG sites and CpG island in the promoters of NAIP and XIAP. The selected CpG island in the NAIP gene promoter is in the region from –634 to –64 bp in the promoter of NAIP. The selected CpG island in the XIAP gene promoter is in the region from –2156 to –1734 bp in the promoter of XIAP. **(B)** DNA methylation status at the CpG island of the promoters of NAIP and XIAP was determined by using MSP. Data are expressed as the percentage of methylation and represent the mean ± SEM of three separated experiments. *p < 0.05. M, methylated DNA, U, unmethylated DNA.

To determine whether ethanol-induced up-regulation of DNMT3a and increase in DNMT activity can result in hypermethylation at the promoters of the IAP genes and whether SFN can diminish ethanol-induced hypermethylation at the promoters of these genes, the methylation status of the CpG islands at the promoters of XIAP and NIAP in hNCCs was analyzed by using methylation-specific PCR (MSP). As shown in Figure 3, exposure of hNCCs to ethanol for 24 h resulted in a significant increase in the methylation levels at the promoters of XIAP and NIAP. Co-treatment with SFN significantly diminished ethanol-induced hypermethylation at the promoters of these anti-apoptotic genes in hNCCs. These results demonstrate that ethanol exposure can induce hypermethylation at the promoters of NAIP and XIAP in hNCCs, which can be prevented by co-treatment with SFN.

### SFN Diminished Ethanol-Induced Down-Regulation of IAP Genes in hNCCs

**FIGURE 4:**
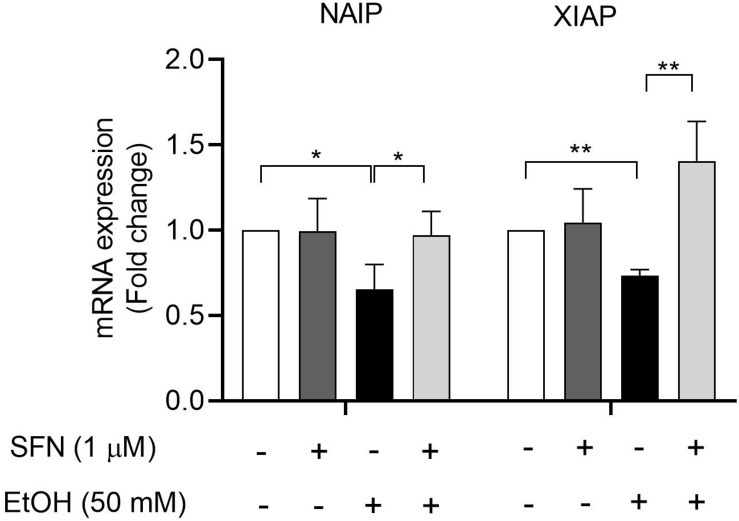
SFN diminished ethanol-induced down-regulation of IAP genes in hNCCs. hNCCs were treated with 1 μM SFN for 24 h, followed by concurrent exposure to 1 μM SFN and 50 mM ethanol for an additional 24 h. The mRNA expression of the NAIP and XIAP was determined by using qRT-PCR. The data were expressed as fold change over control and represent the mean ± SEM of three separated experiments. *p < 0.05, **P < 0.01.

To determine whether ethanol-induced hypermethylation at the promoters of NAIP and XIAP can repress the expression of NAIP and XIAP and whether SFN can prevent ethanol-induced down-regulation of NAIP and XIAP by diminishing ethanol-induced hypermethylation at the promoters of these genes in hNCCs, the mRNA expression of NAIP and XIAP was analyzed in hNCCs. As shown in Figure 4, exposure to 50 mM ethanol for 24 h resulted in a dramatic reduction in mRNA expression of NAIP and XIAP in hNCCs. Treatment with SFN significantly diminished ethanol-induced down-regulation of NAIP and XIAP in hNCCs, indicating that SFN can diminish ethanol-induced repression of NAIP and XIAP in hNCCs by preventing ethanol-induced hypermethylation at the promoters of NAIP and XIAP.

### Knockdown of DNMT3a Significantly Diminished Ethanol-Induced Down-Regulation of NAIP and XIAP and Enhanced the Effects of SFN Against Ethanol-Induced Down-Regulation of NIAP and XIAP in hNCCs

**FIGURE 5:**
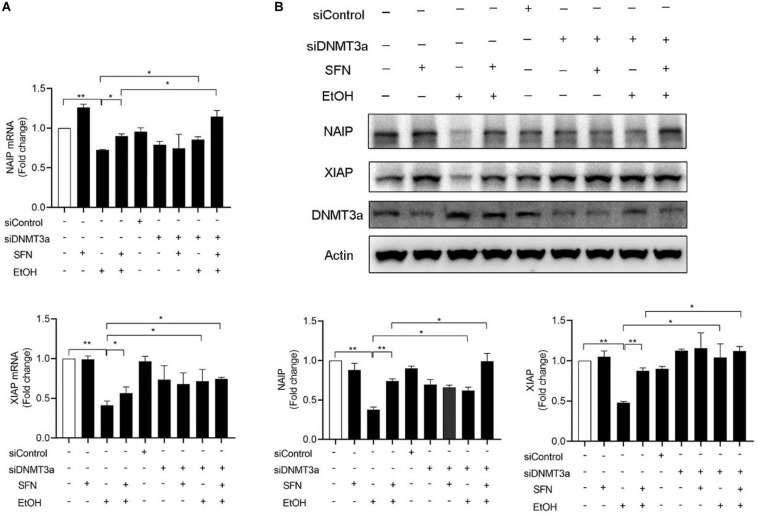
The knockdown of DNMT3a diminished ethanol-induced down-regulation of NAIP and XIAP and enhanced the effects of SFN against ethanol-induced down-regulation of NAIP and XIAP in hNCCs. hNCCs transfected with control-siRNA or DNMT3a-siRNA were treated with 1 μM SFN for 24 h, followed by concurrent exposure to 1 μM SFN and 50 mM ethanol for an additional 24 h. **(A)** The mRNA expression of NAIP and XIAP was determined by qRT-PCR. **(B)** The protein expression of NAIP and XIAP was determined by Western blotting. Data are expressed as fold change over control and represent the mean ± SEM of three separated experiments. *p < 0.05, **p < 0.01.

To determine whether the upregulation of DNMT3a contributes to the ethanol-induced down-regulation of NAIP and XIAP, DNMT3a was knocked down by siRNA in hNCCs before treatment with ethanol or/and SFN. We found that the knockdown of DNMT3a significantly diminished ethanol-induced decreases in the mRNA expression of NAIP and XIAP. Down-regulation of DNMT3a by siRNA also enhanced the effects of SFN against ethanol-induced down-regulation of NIAP and XIAP in hNCCs. In addition, knockdown of DNMT3a significantly diminished ethanol-induced decreases in the protein expression of NAIP and XIAPI and significantly enhanced the effects of SFN against ethanol-induced reductions in the protein expression of NAIP and XIAP (Figure 5). These results indicate that the down-regulation of DNMT3a and the inhibition of DNMT activity by SFN can diminish ethanol-induced repression of anti-apoptotic genes in hNCCs.

### Knockdown of DNMT3a Diminished Ethanol-Induced Apoptosis and Enhanced the Protective Effects of SFN Against Ethanol-Induced Apoptosis in hNCCs

**FIGURE 6:**
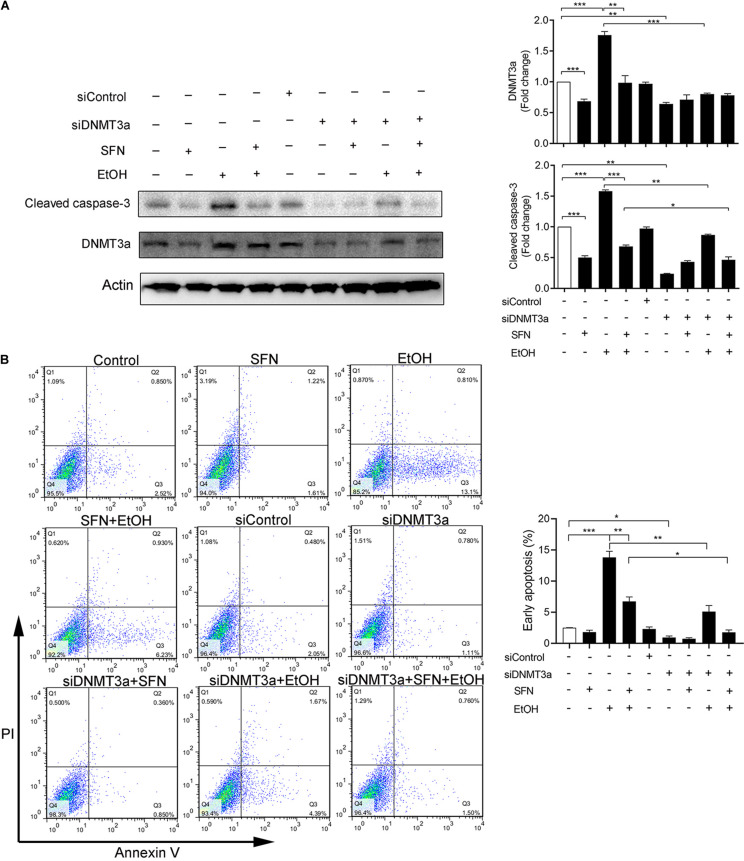
The knockdown of DNMT3a diminished ethanol-induced apoptosis and enhanced the protective effects of SFN in hNCCs. hNCCs transfected with control-siRNA or DNMT3a-siRNA were treated with 1 μM SFN for 24 h, followed by concurrent exposure to 1 μM SFN and 50 mM ethanol for an additional 24 h. The protein expression of DNMT3a was determined by Western blotting **(A)**. Apoptosis was determined by the analysis of caspase-3 cleavage by Western blotting **(A)** or flow cytometry with Annexin V-FITC apoptosis detection kit **(B)**. Data are expressed as fold change over control **(A)** or percentage **(B)** and represent the mean ± SEM of three separated experiments. *p < 0.05, **p < 0.01, ***p < 0.001.

To determine whether ethanol-induced upregulation of DNMT3a and subsequent repression of anti-apoptotic genes, NAIP and XIAP, contributes to ethanol-induced apoptosis and whether SFN can prevent ethanol-induced apoptosis by diminishing ethanol-induced upregulation of DNMT3a, DNMT3a was knocked down by siRNA in hNCCs. Apoptosis was determined by the analysis of caspase-3 activation using Western blot. As shown in Figure 6A, the knockdown of DNMT3a by siRNA significantly reduced ethanol-induced caspase-3 activation, indicating that the knockdown of DNMT3a can diminish ethanol-induced apoptosis in hNCCs. These results were further confirmed by the results from the flow cytometric analysis of Annexin V staining, which have clearly shown that knockdown of DNMT3a can significantly reduce the number of early apoptotic cells in ethanol-exposed hNCCs (Figure 6B). The knockdown of DNMT3a also significantly enhanced the protective effects of SFN against ethanol-induced apoptosis. These results demonstrate that ethanol-induced upregulation of DNMT3a contributes to ethanol-induced apoptosis and that SFN can prevent ethanol-induced apoptosis in hNCCs by diminishing ethanol-induced upregulation of DNMT3a and reducing the activity of DNMTs.

## Discussion

Ethanol-induced apoptosis in NCCs is one of the major mechanisms underlying the pathogenesis of FASD. Previous studies have demonstrated that ethanol exposure during the early stages of development resulted in excessive cell death in NCCs in mouse embryos (Kotch and Sulik, 1992; Dunty et al., 2001). Using an *in vitro* model of mouse NCCs, JoMa1.3, our laboratory has also shown that ethanol exposure significantly increased apoptosis in NCCs (Chen et al., 2015; Li et al., 2019b). In this study, we have shown that exposure to ethanol resulted in a significant increase in apoptosis in hNCCs. This is the first study that demonstrates that ethanol can induce apoptosis in human NCCs.

Apoptosis can be induced and regulated by multiple signaling pathways, including the inhibitor of apoptosis proteins (IAP), a group of negative regulators of apoptosis. IAPs contribute to cell survival by controlling the formation of cell-death-activating platforms, including the apoptosome in Drosophila and the Ripoptosome in mammals, and mediating activation of NF-kB and subsequent induction of pro-survival gene transcription through their ability to functions as E3 ligases (Silke and Meier, 2013). IAPs have been implicated in the pathology of human cancers by inhibiting apoptosis, and the overexpression of IAPs has been found in various cancers (Wright and Duckett, 2005). Studies have also revealed the importance of IAPs in the adaptive response to cell damages (Marivin et al., 2012). It was reported that XIAP and NIAP are involved in the adaptive response of neuronal cells to hypoxic-ischemia-induced injury (Holcik et al., 2000; Russell et al., 2008; West et al., 2009). The down-regulation of XIAP has also been linked to neurodegenerative disorders, including Wilson’s and Huntington’s diseases (Goffredo et al., 2005; Weiss et al., 2010). In addition, IAPs were found to be critical for embryonic development. The knockdown of birc5a resulted in multiple defects in the nervous system, the cardiovascular system, and the hematopoietic system (Delvaeye et al., 2009). In this study, we have shown that ethanol exposure can significantly decrease the expression of NAIP and XIAP and induce apoptosis in hNCCs and that SFN treatment that can diminish ethanol-induced down-regulation of NAIP and XIAP can also decrease the ethanol-induced apoptosis in hNCCs, demonstrating that down-regulation of NAIP and XIAP contributes to ethanol-induced apoptosis in hNCCs.

Epigenetic modifications, including DNA methylation and histone modifications, are well-known mechanisms for the regulation of gene expression and are involved in the regulation of many genes associated with various cellular functions, including apoptosis (Boyanapalli and Kong, 2015; Sui et al., 2015; Kang et al., 2019). Studies have shown that epigenetic modifications contribute to the detrimental consequences of alcohol abuse during pregnancy in the developing fetus (Basavarajappa and Subbanna, 2016). Recent studies in our laboratory have demonstrated that ethanol exposure significantly increased apoptosis through decreasing histone acetylation at the promoters of the anti-apoptotic gene, Bcl2 (Yuan et al., 2018). We have also shown that ethanol-induced reduction in the enrichment of trimethylation of histone H3 Lysine 4 (H3K4me3) at the promoters of Snail1 contributes to ethanol-induced apoptosis in NCCs (Li et al., 2019b). In this study, we have shown that ethanol exposure resulted in a significant increase in the methylation levels at the promoters of XIAP and NIAP and decreased the expression of these anti-apoptotic genes in hNCCs. These results indicate that, in addition to histone modification, DNA methylation also contributes to the regulation of the anti-apoptotic genes in ethanol-exposed NCCs.

DNMTs are key enzymes that catalyze the DNA methylation process by transferring a methyl group to DNA (Robertson et al., 1999; Uysal et al., 2015). DNMT3a is the main enzyme involved in DNA methylation in mammals (Singal and Ginder, 1999). It is well-known that DNMT3a plays a critical role in the establishment of methylation patterns during development (Zahnow et al., 2016). In addition, the defect of DNMT3a was reported to result in non-progressive neurodevelopmental conditions (Weissman et al., 2014). Environmental stimuli in pregnant rat have been found to increase DNMT3a expression and alter DNA methylation levels in their offspring (Jensen Pena et al., 2012). A study has also demonstrated that ethanol exposure increased the expression of DNMT3a in rats (Sakharkar et al., 2014). In addition, it has been reported that DNMT3a can regulate the expression of apoptosis-related genes in cancer cells through changing the methylation levels at the promoter regions of the apoptosis-related genes, such as *Bad, Bax and Bcl2* (Jing et al., 2019; Li et al., 2019a; Wei et al., 2019). Consistent with these findings, we found that ethanol exposure significantly increased the expression of DNMT3a in hNCCs. Ethanol exposure also resulted in a significant increase in the activity of DNMT. In addition, knockdown of DNMT3a dramatically diminished ethanol-induced down-regulation of NAIP and XIAP, demonstrating that the ethanol-induced upregulation of DNMT3a contributes to the hypermethylation at the promoters of NAIP and XIAP and the down-regulation of these anti-apoptotic genes in hNCCs exposed to ethanol.

It has been reported that SFN can act as an inhibitor of histone deacetylase (HDAC) and DNMTs to modulate epigenetic modification of genes in varied types of cells (Ali Khan et al., 2015; Su et al., 2018; Yuan et al., 2018). Studies have demonstrated that SFN can suppress the LPS-induced apoptosis in monocyte-derived dendritic cells through down-regulating DNMT3a and the suppression of TGF-β signaling (Qu et al., 2015). Recent studies from our laboratory have also demonstrated that SFN can reduce ethanol-induced apoptosis in NCCs by diminishing ethanol-induced reduction of histone acetylation at the promoter of Bcl2 and preventing the down-regulation of Bcl2 in ethanol-exposed NCCs (Yuan et al., 2018). In the present study, we found that SFN treatment diminished ethanol-induced upregulation of DNMT3a and dramatically reduce the activity of DNMTs in ethanol-exposed hNCCs. Treatment with SFN also diminished the ethanol-induced hypermethylation at the promoters of NAIP and XIAP and the down-regulation of NAIP and XIAP and subsequently reduced ethanol-induced apoptosis in hNCCs. These results demonstrate that SFN can prevent ethanol-induced apoptosis in hNCCs by epigenetically modulating the DNA methylation at the promoters of anti-apoptotic genes.

In conclusion, the present study has demonstrated that SFN treatment diminished the ethanol-induced upregulation of DNMT3a and dramatically reduced the activity of DNMTs in ethanol-exposed hNCCs. We also found that ethanol exposure induced hypermethylation at the promoter regions of NAIP and XIAP, which were prevented by co-treatment with SFN. SFN also diminished ethanol-induced down-regulation of NAIP and XIAP and apoptosis in hNCCs. The knockdown of DNMT3a significantly enhanced the effects of SFN on preventing the ethanol induced repression of NAIP and XIAP and apoptosis in hNCCs. These results demonstrate that ethanol-induced upregulation of DNMT3a and the hypermethylation at the promoters of NAIP and XIAP and subsequent down-regulation of NAIP and XIAP contribute to ethanol-induced apoptosis in hNCCs and that SFN can prevent ethanol-induced apoptosis in hNCCs by preventing ethanol-induced hypermethylation at the promoters of the genes encoding the IAP proteins. These findings suggest that SFN may represent a promising and effective agent for the prevention of ethanol-induced apoptosis and FASD.

## Data Availability Statement

All datasets generated in this study are included in the article.

## Author Contributions

YL and S-YC conceptualized and designed the experiments and participated in data interpretation and manuscript preparation. YL, HF, and JL performed the experiments and participated in data analysis. FY, LL, WF, and H-GZ participated in data interpretation and discussion. All authors reviewed the manuscript.

## Conflict of Interest

The authors declare that the research was conducted in the absence of any commercial or financial relationships that could be construed as a potential conflict of interest.

## Abbreviations

DNMT: DNA methyltransferase
FASD: Fetal Alcohol Spectrum Disorders
hNCCs: Human neural crest cells
IAP: Inhibitor of apoptosis protein
MSP: Methylation-specific PCR
SFN: Sulforaphane.
